# Catalogue of Skulls of Man and the Inferior Animals, in the Collection of Samuel George Morton, M. D., Penn, and Edinb., President of the Academy of Natural Sciences of Philadelphia, &c. &c.

**Published:** 1850-02

**Authors:** 


*Catalogue of Skulls of Man and the Inferior Animals, in the collection of* Samuel George Morton, M. D., Penn. and Edinb. President of the Academy of Natural Sciences of Philadelphia, &c., &c. pp. 84.

It was said of Sydenham, that if, in any one way, he differed from other men, it was in his power of continued attention—of faithful, unbroken observation. "It requires more strength and firmness of mind," says his reviewer, "more of what deserves to be called genius, to make a series of genuine observations on medicine, or any other art, than to spin any number of nice hypotheses." This justly applies to the industrious author of the work before us. For twenty years has he been engaged in collecting from every source the materials of this catalogue, (upon which, also, are based his well known works, "Crania Ægyptiaca," and "Crania Americana.") His cabinet, which forms a striking feature in the Museum of the Academy of Natural Sciences of Philadelphia, now contains 1468 crania, of which 867 are human, and the remainder those of the inferior animals.

The primary motive of the author was a comparison of the crania in the different races of men, and these again with those of the inferior animals; not only in reference to the exterior form, but also to internal capacity, as indicative of the size of the brain. Besides these strictly Ethnographic objects, the pathological condition of the skull from diseases and wounds, as well as remarkable developments illustrative of the principles of Phrenology and preternatural growth of every description, were prominent in the author's mind.

The following analysis exhibits an Ethnographic view of the materials contained in the entire series.

I. *Caucasian Race.*—Anglo-American, 8; Anglo-Irish, 1; Armenian, 6; Affghan, 1; Arab, 3; Arab-Egyptian or Fellah, 19; Celtic Irish, 5; Circassian, 4; Dutch, 1; English, 5; Egyptian, Ancient 84; Græco-Egyptian or Pelasgic, 22; Guanché, 1; German, 13; Hindu, 35; Midianite, 1; Parsee, 2; Phœnician, 1; Prussian, 4; Swede, 2; Semitic-Egyptian, 7; Uncertain, 1.

II. *Mongolian Race.*—Chinese, 7; Laplander, 1.

III. *Malay Race.*—Amboynese, 3; Bornese, 2; Ballinese, 1; Cingalese, 1; Javanese, 5; Kanaka, 7; Makassar, 3; Madurese, 2; Malakese, 1; New Zealanders, 3; Sambawese, 1; Tagelos, 1; Various, 4.

IV. *Aboriginal American Race.*—Assinaboin, 3; Araucanian, 7; Aztec, 2; Arickaree, 3; Chechemecan, 1; Cotonay, 3; Cayuga, 1; Cherokee, 5; Chetimaches, 2; Creek, 2; Choctaw, 1; Charib, 2; Chippeway, 2; Chimuyan, 1; Chemesyan, 1; Chenouk, 7; Cowalitsk, 1; Clatsap, 1; Clickitat, 1; Chayenne, 1; Dacota, 1; Euchee, 1; Gepepscot, 1; Huron, 5; Illinois, 2; Iroquois, 2; Kalapooyah, 1; Klatstoni, 1; Lenape, 3; Lipan, 2; Mexican, 12; Manta, 1; Mandan, 7; Minetari, 4; Missouri, 1; Mingo, 1; Miami, 6; Mohawk, 3; Menominee, 7; Massasauga, 1; Maya, 1; Mound and cave skulls, 17; Natchez, 1; Natick, 1; Naumkeag, 1; Narraganset, 10; Nanticoke, 1; Ottomie, 5; Ottigamie, 3; Oneida, 1; Osage, 2; Otoe, 4; Ottowa, 4; Peruvian, 201; Pawnee, 2; Potowatomie, 3; Pames, 2; Quichua, 1; Quinnipiak, 1; Seminole, 15; Sauk, 3; Shawnee, 4; Shoshone, 3; Tlahuica, 1; Tlascalan, 1; Upsarooka, 2; Winnebago, 2; Yamassee? 3; Uncertain tribes, 5.

V. *Negro Race.*—Native African Negroes:—Benguela, 1; Bassa, 3; Congo, 1; Grabbo, 1; Mina, 1; Mozambique, 2; Kroo, 2; Eboe, 2; Golah, 2; Pessah, 3; Dey, 2; Macua, 1; Various, 57. Negroes born in America, 12; Embalmed Negro, 1; Caffer. 1; Hottentot, 3; Australian, 8; Oceanic Negro, 1; Hovah, 2; Tashmanian? 1.

VI. *Mixed Races.*—Copt, Modern, 3; Hispano-Peruvian, 2; Mulatto, 2; Negro and Indian, 2; Negroid Egyptian, 11; Copt, Ancient, 2; Modern Nubian? 1; Uncertain, 2.

VII. *Lunatics.*—Anglo-American, 2; English, 1; German, 1; Irish, 1; Mulatto, 2; Negro, 2.

VIII. *Idiots.*—Anglo-American, 1; Dutch, 1; European, 1; Ancient Egyptian, 2; Malay, 1; Negro, 1.

Skulls illustrative of growth, 7; Skulls illustrative of disease, 9; Casts of skulls, 26.

The measurements of the internal capacity of the crania mentioned in this catalogue are the result of the labor of the author's own hands, and have been restricted to those of individuals of sixteen years of age and upwards, at which period the brain is believed to possess the adult size. The method of obtaining the internal capacity is singularly accurate, a hundred measurements not varying a single inch. It consists in substituting leaden shot, ⅛ of an inch in diameter, for the white mustard seed originally used. The foramina are stopped, and the cavity of the cranium filled with shot through the foramen magnum, until, by shaking and compressing, it will contain no more. The shot are then transferred to an accurate measure prepared for the measurement of cubic inches, by which the internal capacity is accurately marked. The following table expresses the size of the brain in cubic inches, as obtained from the measurement of 623 crania of various races and families of men.

"TABLE,*

*Showing the Size of the Brain in cubic inches, as obtained from the internal measurement af* 623 *Crania of various Races and Families of Man.*

| RACES AND FAMILIES. | No. of Skulls. | Largest I. C. | Smallest I. C. | Mean. | Mean. |
|---|---|---|---|---|---|
| MODERN CAUCASIAN GROUP. | | | | | |
| TEUTONIC FAMILY. | | | | | |
| *Germans,* | 18 | 114 | 70 | 90 | 92 |
| *English,* | 5 | 105 | 91 | 96 | |
| *Anglo-Americans,* | 7 | 97 | 82 | 90 | |
| PELASGIC FAMILY. *Persians, Armenians, Circassians,* | 10 | 94 | 75 | 84 | |
| CELTIC FAMILY. *Native Irish,* | 6 | 97 | 78 | 87 | |
| INDOSTANIC FAMILY. *Bengalees, &c.,* | 32 | 91 | 67 | 80 | |
| SEMITIC FAMILY. *Arabs,* | 3 | 98 | 84 | 89 | |
| NILOTIC FAMILY. *Fellahs,* | 17 | 96 | 66 | 80 | |
| ANCIENT CAUCASIAN GROUP. | | | | | |
| From the Catacombs. PELASGIC FAMILY. *Græco-Egyptians,* | 18 | 97 | 74 | 88 | |
| From the Catacombs. NILOTIC FAMILY. *Egyptians,* | 55 | 96 | 68 | 80 | |
| MONGOLIAN GROUP. | | | | | |
| CHINESE FAMILY. | 6 | 91 | 70 | 82 | |
| MALAY GROUP. | | | | | |
| MALAYAN FAMILY. | 20 | 97 | 68 | 86 | 85 |
| POLYNESIAN FAMILY. | 3 | 84 | 82 | 83 | |
| AMERICAN GROUP. | | | | | |
| TOLTECAN FAMILY. *Peruvians,* | 155 | 101 | 58 | 75 | 79 |
| *Mexicans,* | 22 | 92 | 67 | 79 | |
| BARBAROUS TRIBES. *Iroquois, Lenapé, Cherokee, Shoshoné, &c.,* | 161 | 104 | 70 | 84 | |
| NEGRO GROUP. | | | | | |
| NATIVE AFRICAN FAMILY. | 62 | 99 | 65 | 83 | 83 |
| AMERICAN-BORN NEGROES. | 12 | 89 | 73 | 82 | |
| HOTTENTOT FAMILY. | 3 | 83 | 68 | 75 | |
| ALFORIAN FAMILY. *Australians,* | 8 | 83 | 63 | 75 | |

*The letters I. C. designate the internal capacity.

In this table the measurements of children, idiots and mixed races are omitted, excepting only in the instance of the Fellahs of Egypt, who, however, are a blended stock of two Caucasian nations,—the true Egyptian and the intrusive Arab, in which the characteristics of the former greatly predominate.

No mean has been taken of the Caucasian race* collectively, because of the very great preponderance of Hindu, Egyptian and Fellah skulls over those of the Germanic, Pelasgic and Celtic families. Nor could any just *collective* comparison be instituted between the Caucasian and Negro groups in such a table, unless the small-brained people of the latter division (Hottentots, Bushmen and Australians) were proportionate in number to the Hindoos, Egyptians and Fellahs of the other group. Such a computation, were it practicable, would probably reduce the Caucasian average to about 87 cubic inches, and the Negro to 78 at most, perhaps even to 75, and thus confirmatively establish the difference of at least nine cubic inches between the mean of the two races.

The wide-spread reputation of the author, and his habits of close observation, entitle him to the fullest confidence on this subject, and we are pleased to learn that he is engaged in a memoir which will embrace detailed conclusions from the data collected by his untiring zeal, and which we predict will still further extend his fame as an Ethnologist.

* It is necessary to explain what is here meant by the word *race.* Further researches into Ethnographic affinities will probably demonstrate that what are now termed the *five races* of men, would be more appropriately called *groups;* that each of these groups is again divisible into a greater or smaller number of primary races, each of which has expanded from an aboriginal nucleus or centre. Thus I conceive that there were several centres for the American group of races, of which the highest in the scale are the Toltecan nations, the lowest the Fuegians. Nor does this view conflict with the general principle, that all these nations and tribes have had, as I have elsewhere expressed it, a common origin; inasmuch as by this term is only meant an indigenous relation to the country they inhabit, and that collective identity of physical traits, mental and moral endowments, language, &c., which characterize all the American races. The same remarks are applicable to all the other human races; but in the present infant state of Ethnographic science, the designation of these primitive centres is a task of equal delicacy and difficulty. I may here observe, that whenever I have ventured an opinion on this question, it has been in favor of the doctrine of *primeval diversities* among men,—an original adaptation of the several races to those varied circumstances of climate and locality, which, while congenial to the one are destructive to the other; and subsequent investigations have confirmed me in these views. See *Crania Americana*, p. 3; *Crania Ægyptiaca*, p. 37; *Distinctive Characteristics of the Aboriginal Race of America*, p. 36; *Silliman's American Journal of Science and the Arts*, 1847; and my Letter to J. R. Bartlett, Esq. in Vol. 2 of the Transactions of the Ethnological Society of New York."

